
JOURNAL INFORMATION
==============================
NLM Title Abbreviation: Atlanta Med Surg J (1884)
NLM Journal ID: 101251623

Atlanta Medical and Surgical Journal (1884)

Publisher: Biomedical Journal Digitization Project

ARTICLE INFORMATION
==============================
PMCID: PMC9201840
Article version: 1
Subjects: Book Reviews, Pamphlets, Exchanges

Book Reviews

Print publication date: 1899 Jan
Volume: 15
Volume Series: New Ser
Issue: 11
First page: 766
Last page: 770
License: This work is free of known copyright restrictions. For more information, please see PMC Back Issue Digitization.
License URL: https://creativecommons.org/publicdomain/mark/1.0/

==============================

### *BOOK REVIEWS, PAMPHLETS, EXCHANGES.*

### *BOOK REVIEWS.*

[Contributions solicited for review.]

Human Anatomy. A Complete Systematic Treatise. By various authors. Including a special section on Surgical and Topographical Anatomy. Edited by Henry Morris, M.A. and M.B., London, Senior Surgeon to Middlesex Hospital; Examiner in Surgery in the University of London; Member of the Council, and Chairman of the Court of Examiners of the Royal College of Surgeons of England; Honorary Member of the Medical Society of the County of New York. Illustrated by seven hundred and ninety wood-cuts, the greater part of which are original and made expressly for this work by a special artist. Over 200 printed in colors. Second edition, revised and enlarged. P. Blakiston's Son & Co., 1012 Walnut Street, Philadelphia, 1898. Price, $6.00.

We are glad to see that this book is gradually gaining favor in this country, for the texts are concise and plain.

This edition has been completely revised, and many new cuts added. Many of these are photographed from original dissections in the museum of the Royal College of Surgeons, the Hunterian Museum and the museum of St. Bartholomew's Hospital. These cuts have the advantage over those made diagrammatically, in that the student can get a better idea of the relation of the different structures. A special feature, too, is the mode of coloring the cuts, the origin of muscles being marked in red lines, insertion in blue and ligaments in dotted black lines. The description of these cuts printed at the end of the pointers is in different types for the different structures, so one can see what one wants at a glance.

A short space is also devoted to the development of bones; the epiphyseal lines of the long bones are shown in wood-cuts, made specially for this book.

The articles on each section of the body have been written by men eminently fitted for the work assigned them. While each is personally responsible for the part assigned him, the articles have been read by one or two of the other authors, so that it may be considered that each article has received their approval.

J. L. C.

---

Practical Urinalysis and Urinary Diagnosis: A Manual for the Use of Physicians, Surgeons, and Students. By Charles W. Purdy, M.D., LL.D. (Queen's University); Fellow of the Royal College of Physicians and Surgeons, Kingston; Professor of Clinical Medicine at the Chicago Post-Graduate Medical School. Author of "Bright's Disease and Allied Affections of the Kidneys"; also of "Diabetes: Its Causes, Symptoms, and Treatment." Fourth revised edition. With numerous illustrations, including Photo-engraving and Colored Plates. In One crown octavo volume, 365 pages, bound in extra cloth, $2.50 net. The F. A. Davis Co., Publishers, 1914–16 Cherry St., Philadelphia; 117 W. Forty-second St., New York City; 9 Lakeside Building, 218–220 S. Clark St., Chicago, Ill.

This excellent work has now reached its fourth edition. As a practical work on Urinalysis and Urinary Diagnosis, it is probably one of the best in the English language. It is thoroughly up to date, and each portion of the work is exceedingly clear and concise. The author has divided the book into two parts. Part I. Analysis of Urine. In this is treated General Consideration as to Color, etc., of Urine; Composition of Normal Urine; Proteids; Carbohydrates; Abnormal Urine; Urinary Sediments; Anatomical Sediments; Gravel and Calculus. Part II. Urinary Diagnosis, Treating of Diseases of the Urinary Organs and Urinary Disorders; Urine in other Diseases; Examination of Urine for Life Insurance. It is a most excellent work.

---

A Text-Book of Pathology. By Alfred Stengel, M.D., Instructor in Clinical Medicine in the University of Pennsylvania, etc. Published by W. B. Saunders, Philadelphia. Price, Cloth, $4.00; Half Morocco, $5.00.

Dr. Stengel has given us a most excellent work on Pathology, and as a text-book on this subject it must commend itself to all teachers. The work is from the pen of a practical clinical teacher, and as such shows these landmarks throughout its pages. The author goes well into the minute microscopical details of various pathological conditions, and yet, he gives just what is needed, and nowhere is there a mark of verbosity. In his preface the author states, "except in a few instances, discussion of methods of examination has been omitted, because it seemed unwise to increase the size of the book with matter that is appropriately presented in special works on technique. For similar reasons the author has decided to exclude pathology of the skin and the organs of special sense." The author was, no doubt, wise in this arrangement, for as it is the book is sufficiently large, just as it is. The work is divided into Part I., General Pathology, and Part II., Special Pathology. The illustrations are good, and the cuts beautifully executed. In fact, these latter make the most attractive features of the book, which is, indeed, an important item.

---

KING'S AMERICAN DISPENSATORY. New edition. Entirely rewritten and enlarged. By Harvey W. Felter, M.D., Adjunct Professor of Chemistry in the Eclectic Medical Institute, Cincinnati, O.; Co-editor Locke's Materia Medica aud Therapeutics; President Ohio State Eclectic Medical Association, etc.; and John Uri Lloyd, Ph.M., Professor of Chemistry and Pharmacy in the Eclectic Medical Institute, Cincinnati, O.; formerly Professor of Pharmacy in the Cincinnati College of Pharmacy; Ex-President of the American Pharmaceutical Association; Author of the Chemistry of Medicines; Drugs and Medicines of North America; Etidorhpa; etc. Two volume edition, royal octavo, each volume containing over 950 pp. with complete indexes. Cloth, $4.50 per volume post-paid. Sheep, $5.00 per volume post-paid. Volume I. now ready. The Ohio Valley Co., Publishers, Cincinnati, O.

The first volume of King's Dispensatory has been received. This volume is exceedingly well gotten up and well arranged. The various subjects contained in a dispensatory are clearly and accurately described, and it is a work which will prove of great value in the Pharmaceutical Laboratory. We congratulate the authors.

THE CARE OF THE BABY. By I. D. Crozier Griffeth, M.D.

This book is a desideratum long needed. Many books have been written purporting to give the information this does, but as a rule by men unfit either by experience, medical knowledge or scientific attainment to do justice to the subject, and its precepts often doing harm instead of good.

The author of this book is fully competent for the task, and has given one that, from its simplicity of language and want of technical phraseology, cannot only be understood and appreciated by every intelligent mother and nurse, but gives facts and lessons well worthy the study of physicians—old, middle-aged and young.

It should be a text-book in every medical college where the life and health of children are considered, and that should be done in every college.

He takes the baby in utero and carries it through all of its periods of existence until it is able to take care of itself.

Every young woman expecting to become a mother should read it carefully. She will find in its pages much valuable information she never dreamed of, observance of which will calm much of the anxiety and dread of which she is possessed and teach her how to conduct herself so as to avoid unnecessary pain and danger. To the monthly nurse the book is invaluable. It gives rules for the management of the mother during confinement and until she is safely through this ordeal, besides full and explicit directions for the care of the child in all of its details until able to care for itself. He gives much attention to the care of the sick baby and lays down many simple but excellent rules for its management, both as preventive and curative suggestions and aids.

I very cordially recommend "The Care of the Baby" to mothers, nurses and physicians.

It is published and for sale by W. B. Saunders, 925 Walnut St., Philadelphia. G. G. R.

HISTOLOGY, NORMAL AND MORBID. By Edward K. Dunham, M.D., Professor of General Pathology, Bacteriology, and Hygiene, in the University and Bellevue Hospital Medical College, New York. In one very handsome octavo volume of 448 pages, with 363 illustrations. Cloth, $3.25, net. Lea Brothers & Co., Publishers, Philadelphia and New York.

As is stated in the preface to this book, the author intends it more as an elementary work than an exhaustive treatise on the subject of normal and morbid histology. As such the author has succeeded most admirably, and the student will find it a most excellent ready reference book for the library. The illustrations are all that could be desired, and they are taken from other standard works or monographs, thus greatly enhancing the value of the book. The reproduction of these different cuts has been most beautifully executed, and we must admire the excellency of the same. Professor Dunham is to be congratulated upon the production of this book.

---

HYGIENE OF THE VOICE. By Thos. F. Rumbold, M.D., of St. Louis, Mo. Published by Witt Publishing Co., St. Louis.

This is a most excellent little book, neatly gotten up by a man well known as a laryngologist. It is clear and practical. While not written especially for medical men, it nevertheless contains a great deal of useful information to such. The author says in his preface as to the contents of the book: "No medical treatment is recommended, except a few simple remedies for temporary relief, and those only of such a character as would not, under any circumstance, be harmful, however taken." He says further, which is every bit true: "My long experience teaches me that none but medical men should attempt the treatment of voice-users." This little brochure will prove of special value to public singers and speakers.
